# Case report: Visual acuity loss as a warning sign of ocular syphilis: A retrospective analysis of 17 cases

**DOI:** 10.3389/fmed.2022.1037712

**Published:** 2022-10-17

**Authors:** Yating Xu, Jingjing Li, Yuxuan Xu, Wei Xia, Xingfan Mo, Mingzhi Feng, Fanghua He, Shanshan Li, Fangzhi Du, Qianqiu Wang, Minzhi Wu

**Affiliations:** ^1^Department of Dermatology, The Fifth People's Hospital of Suzhou, The Affiliated Hospital of Infectious Diseases of Soochow University, Suzhou, Jiangsu, China; ^2^Ophthalmology Department, The First Affiliated Hospital of Soochow University, Suzhou, Jiangsu, China; ^3^Institute of Dermatology, Chinese Academy of Medical Sciences and Peking Union Medical College, Nanjing, Jiangsu, China

**Keywords:** ocular syphilis, neurosyphilis, visual acuity loss, uveitis, stromal keratitis

## Abstract

**Objectives:**

To define the clinical features of ocular syphilis and analyze the cerebrospinal fluid (CSF) of ocular syphilis patients to determine the co-occurrence of neurosyphilis.

**Methods:**

This was a retrospective study of 17 patients (23 eyes) with ocular syphilis admitted to the Fifth People's Hospital, Suzhou, China from September 2017 to December 2021. Clinical manifestations, laboratory tests, treatment, and clinical outcomes were analyzed, and a review was conducted.

**Results:**

Eight males (12 eyes) and nine females (11 eyes) were enrolled. Mean patient age was 49.06 ± 3.47 years. The total manifestation time for ocular symptoms ranged from 10 days to 6 years. The cohort was comprised of three cases of early syphilis, four cases of late syphilis, and ten cases of unknown stage. The primary complaints were decreased visual acuity in 15 cases (21 eyes), ptosis in 1 case (1 eye), and loss of light perception in 1 case (1 eye). Cases were diagnosed as chorioretinitis in 7 cases (8 eyes), optic nerve retinitis in 4 cases (6 eyes), optic neuritis in 4 cases (7 eyes), and oculomotor nerve palsy in 1 case (1 eye), syphilitic stromal keratitis in 1 case (1 eye). Serum HIV antibody was positive in one case(Nos.2). All patients had reactive serum Treponema Pallidum Particle Agglutination (TPPA) and Toluidine Red Unheated Serum Test (TRUST). All patients underwent CSF examination. CSF white blood cell count was ≥5 × 10^6^/L in 13 cases, CSF protein was >500 mg/L in 6 cases, TPPA was reactive in 15 cases, and TRUST was reactive in 5 cases. Eleven cases were also diagnosed with neurosyphilis. Patients were treated with either penicillin G sodium or ceftriaxone sodium. At time of discharge, 12 patients reported improved visual acuity. Abnormal serum or CSF examination improved in ten patients during the 6–12 month follow-up.

**Conclusion:**

Visual acuity loss is a warning indicator of ocular syphilis. Ocular syphilis primarily manifests as posterior uveitis, involving the choroid, retina, and optic nerve, and often co-occurs with neurosyphilis. Effective treatment should be administered immediately to avoid irreversible visual impairment and other serious adverse outcomes.

## Introduction

Syphilis is a chronic and systemic venereal disease caused by *Treponema pallidum* infection. *Treponema pallidum* can affect multiple organs of the human body and any part of the eye, manifesting as ocular syphilis. The Centers for Disease Control and Prevention (CDC) define ocular syphilis as “clinical symptoms or signs consistent with ocular disease such as uveitis, panuveitis, diminished visual acuity, blindness, optic neuropathy, interstitial keratitis, anterior uveitis, or retinal vasculitis accompanying syphilis of any stage (1)”. Recently, as the incidence of syphilis has increased, reports of ocular syphilis have correspondingly increased. In diagnosed syphilis, 0.53 and 0.65% of patients had comorbid suspected ocular syphilis in 2014 and 2015, respectively, in the United States (2). Of 3,678 syphilis patients in British Columbia from 2013–2016, 1.1% had ocular manifestations (3). The incidence of ocular syphilis in the United Kingdom was 0.3 cases per 1,000,000 people, and 0.6% of syphilis patients had ocular symptoms (4). A 6 year case study in eastern China demonstrated that ocular syphilis was comorbid in 5.1% of total syphilis cases (5). In Shanghai, 213 (2.6%) of 8,310 syphilitic patients had ocular manifestations from October 2009 to October 2017 (6).

Early and effective treatment is imperative to prevent the incidence of ocular syphilis and serious potential consequences. Through clinical observation of cases of ocular syphilis cases, we identified that visual acuity loss was the primary manifestation in all cases, suggesting this manifestation could be used as a warning indicator for ocular syphilis. Clinical data of 17 cases of ocular syphilis are reported as follows.

### Clinical data

Clinical data of 17 patients with ocular syphilis admitted to The Fifth People's Hospital of Suzhou, Jiangsu Province from September 2017 to December 2021 were retrospectively analyzed. Patients' medical history, clinical manifestations, syphilis serological test, cerebrospinal fluid test, diagnosis, treatment, and clinical outcomes were reviewed. Inclusion criteria included the following: (1) according to CDC guidelines (7), positive serum toluidine red unheated serum test (TRUST) and serum Treponema pallidum particle agglutination test (TPPA). (2) ocular manifestations indicative of ocular syphilis. Exclusion criteria included (1) patients with eye diseases of other etiologies such as hypertension, diabetes, and other systemic diseases or autoimmune diseases, and (2) patients with congenital eye disease. The diagnostic criteria for neurosyphilis were (8) (1) epidemiological history, including unprotected sex and history of syphilis infection or infection of sexual partners. (2) presence or absence neurological clinical manifestations. (3) positive serological non-treponema test (RPR, TRUST, or VDRL) positive and serological Treponema test (TPPA or TPHA). (4) CSF white blood cell count ≥5 × 106/L (> 20 × 10^6^ in HIV-positive patients) and/or CSF protein was >500 mg/L, CSF-TRUST, and/or positive TPPA. Other causes of CSF abnormalities were excluded.

## Methods

The study was approved by the Ethics Committee of the Fifth People's Hospital of Suzhou ([(2020) No. 034]). A total of 17 ocular syphilis cases were examined by an ophthalmologist/ophthalmologic expert. Patient sera and CSF samples were tested, as part of the clinical work up. Ophthalmic function tests included visual acuity, pupillary light response test, eye movement test, visual field, visual evoked potential (VEP). Ophthalmic morphological examinations included slit lamp examination and fundus examination. Ophthalmic imaging examinations were conducted using ocular B-ultrasound, fundus fluorescein angiography (FFA), optical coherence tomography (OCT), magnetic resonance imaging. Ophthalmic laboratory tests included collection of intraocular fluid (aqueous humor) for syphilis-related antibody testing. Blood tests included routine CBC-chem, serum biochemical, T-cell subsets, hepatitis B, hepatitis C, HIV, TRUST, and TPPA. CSF tests included CSF routine, CSF biochemical, CSF-TRUST, and CSF-TPPA. All patients were diagnosed with ocular syphilis and hospitalized. The patients were treated with the recommended regimen for ocular syphilis (8), intravenous infusion of penicillin (4 million units) every 4 h for 14 consecutive days, followed by intramuscular injection of benzathine penicillin (2.4 million units) weekly for 3 weeks. Patients with penicillin allergy received ceftriaxone (2 g) intravenously once daily for 14 consecutive days, and the absence of ceftriaxone allergy was confirmed. All patients received oral corticosteroids to alleviate the Jarisch-Herxheimer reaction. Glucocorticoid eye drops or oral corticosteroids were used to decrease the inflammatory response to eye injury, and patients with optic nerve damage received neurotrophic drugs, such as mecobalamine.

## Results

Twenty-three eyes of seventeen patients were enrolled (8 males, 12 eyes; 9 females, 11 eyes). Unilateral eye involvement was present in 11 cases, and bilateral involvement was present six cases. Mean patient age was 49.06 ± 3.47 years (range, 27–74 years), and median age was 51 years. The course of ocular symptoms lasted from 10 days to 6 years. The cohort included three cases of early syphilis, one case of secondary syphilis, four cases of late syphilis, and ten cases of unidentified disease stage. Seven patients (Nos. 6, 7, 8, 11, 15, 16, and 17) were previously misdiagnosed as optic neuroretinitis caused by other causes and did not improve after treatment. Prior to admission, four patients (Nos. 1, 4, 9, and 16) received three weekly intramuscular injections of benzathine penicillin (2.4 million units), and two patients (Nos. 5 and 7) received one intramuscular injection of benzathine penicillin (2.4 million units) once before admission in an attempt to treat syphilis. The other 11 cases were all diagnosed with syphilis for the first time upon admission and had no definite history of syphilis infection or antisyphilitic treatment.

### Ocular manifestations

Fifteen cases (21 eyes) presented with visual acuity loss only, 1 case (1 eye) presented with ptosis, and 1 case (1 eye) presented with blindness.

### Ophthalmic examination

Two patients underwent anterior segment examination. One case (one eye) exhibited local grayish-white opacity of the corneal stroma, and one case (one eye) exhibited corneal clouding. Fundus examination of five patients revealed a leopard striated fundus in one case (one eye) and yellow-white macular lesions in two cases (three eyes). Argyll-Robertson pupil was detected in two cases (three eyes), and ptosis was detected in one case (one eye). Perimetry identified one case (one eye) with visual field defect. FFA examination of four cases revealed chorioretinitis in two cases (three eyes), branch retinal vein occlusion in one case (two eyes), and vitreous opacity with retinal vasculitis in one case (one eye). Two patients underwent OCT examination, revealing retinal lesions in one case (two eyes) and corneal stromal layer involvement in one case (one eye). Four cases (eight eyes) had abnormal intraocular ultrasound. Detection of serum and aqueous humor antibodies revealed that the *Treponema pallidum* antibody was positive, and that the Goldmann-Witmer coefficient (GWC), which calculated as (*T. pallidum* IgG titer in aqueous humor/total IgG titer in aqueous humor)/(*T. pallidum* IgG titer in serum/total IgG titer in serum) (9) was increased in one case (one eye). MRI examination revealed optic atrophy in two cases (four eyes) (Table 1).

**Table 1:** Ocular examination and manifestations of 17 ocular syphilis patients.

**Case**	**Clinical manifestations**	**Ophthalmic examination**								**Ophthalmological diagnosis**	**post-treatment visual acuity**
		**Anterior segment**	**Fundus**	**FFA**	**OCT**	**Ophthalmic ultrasound**	**Aqueous humor detection**	**MRI**	**pre-treatment Visual acuity**		
1	Visual acuity loss in both eyes	—	—	—	—	—	—	Bilateral optic nerve atrophy	VOD:0.25, VOS:0.1	Optic neuritis in both eyes	VOD:0.15, VOS:0.15
2	Visual acuity loss in left eye	—	—	Vitreous opacity of the left eye with retinal vasculitis	—	—	—	—	VOD:0.12, VOS:digit/in front of eye	Chorioretinitis in left eye	VOD:0.12,VOS: 0.06;Decreased vitreous opacity of the left eye
3	Visual acuity loss in both eyes	—	—	—	—	—	—	—	VOS: 1.0, VOD: 0.8	Retinitis of the optic nerve in both eyes	VOD:1.5;VOD: 1.2; Partial decrease of papilledema in both eyes
4	Visual acuity loss in right eye	—	Leopard striated fundus in right eye	—	—	Abnormal ultrasound in both eyes	—	—	—	Chorioretinitis in right eye	-
5	Visual acuity loss in right eye	—	Macular yellow-white lesions in right eye	—	—	—	—	—	VOD:0.1, VOS:0.8	Chorioretinitis in right eye	VOD:0.6,VOS:0.8, Macular edema resolved
6	Visual acuity loss in left eye	—	—	—	—	Punctate weak echoes visible in the vitreous cavities of both eyes	—	—	—	Chorioretinitis in left eye	-
7	Visual acuity loss in both eyes	—	—	Chorioretinitis in both eyes	—	Abnormal ultrasound in left eye	—	—	VOD:0.5, VOS:0. 2	Chorioretinitis in both eyes	VOD:0.25,VOS:0.15
8	Visual acuity loss in both eyes	—	—	Branch retinal vein occlusion in right eye, retinal vasculitis in both eyes	—	Patchy and filamentous ultrasound in both eyes	—	—	VOD:digit/20 cm	Retinitis of the optic nerve in both eyes	VOD:0.2,VOS:0.4; Papilledema was reduced in both eyes
9	Visual acuity loss in left eye	—	—	Chorioretinitis in left eye	—	—	—	—	VOD:0.2;VOS:digit/ 20 cm	Chorioretinitis in the left eye	VOD:0.8,VOS:0.3; Inflammation significantly decreased in left eye
10	Visual acuity loss in both eyes	—	Argyll-Robertson pupils in both eyes	—	—	—	—	Bilateral optic nerve atrophy	VOD:0.15,VOS:0.15	Optic neuritis in both eyes	VOD:0.12;VOS:0.15
11	Blindness in left eye	—	Visual field defect	—	—	—	—	—	VOD:1.0; VOS: light sensitive	Optic neuritis in the left eye	VOD:1.0; VOS: digit
12	Visual acuity loss in left eye	—	—	—	—	—	—	—	—	Chorioretinitis in the left eye	-
13	Blurred vision in right eye accompanied by distension and discomfort	Local grayish-white opacity of corneal stroma of right eye	—	—	Involvement of the corneal stromal layer in the right eye	—	Goldmann-Witmer coefficient 356.26	—	VOD:0.3,VOS:0.25	Stromal keratitis of the right eye	VOD:0.6,VOS:0.6; Decreased corneal stromal opacity in right eye
14	Visual acuity loss in right eye	—	Yellow-white macular lesions in right eye	—	—	—	—	—	VOD:0.25,VOS:1.0	Chorioretinitis in the right eye	VOD:0.6;VOS:1.0; Resolution of the macular lesion
15	Visual acuity loss in left eye	Clouded cornea in left eye	—	—	—	—	—	—	VOD 0.5,VOS 0.08	Retinitis in the left eye	VOD 0.5,VOS:0.15
16	Visual acuity loss in both eyes	—	—	—	Retinal involvement	—	—	—	—	Optic neuritis in both eyes	-
17	Blepharoptosis in right eye	—	Argyll-Robertson pupils and ptosis in the right eye	—	—	—	—	—	—	Oculomotor nerve palsy in the right eye	-

### Laboratory tests

One case was HIV-positive. All cases had positive serum TPPA and TRUST. Three patients had TRUST titers <1:32 and 14 patients were ≥1:32. CSF analysis identified 13 cases with white blood cell count ≥5 × 10^6^/L, six cases with protein >500 mg/L, 15 CSF TPPA-positive cases, five CSF TRUST-positive cases (one case with titer 1:1, two cases 1:4, one case 1:16, one case 1:32). Neurosyphilis was probable in 3 cases (Nos. 3,9,11)and definited in 8 cases(Nos. 1,4,8,10,12,13,14,16). One patient (No. 16) had the characteristic neurosyphilis manifestation of memory loss (see Table 2).

**Table 2:** Laboratory test results and ophthalmological diagnosis of 17 ocular syphilis cases.

**Case**	**Age (y)/ sex**	**Course of disease**	**HIV**	**Serum**		**Cerebrospinal fluid**				**Stages of syphilis**	**Neuro syphilis (+/-)**	**Other symptoms of syphilis**
				**TRUST**	**TPPA**	**Cells**	**Albumin**	**TRUST**	**TPPA**			
						**(10^6^/L)**	**n(mg/L)**					
1	51/M	1 y	-	1:8	+	8	620	-	+	Early stage	+	-
2	31/M	1 m	+	1:256	+	11	391	-	+	undefined	-	-
3	45/F	20 d	-	1:256	+	15	296	-	+	undefined	+	-
4	55/F	4 m	-	1:64	+	12	305	1:1	+	undefined	+	-
5	36/M	10 d	-	1:32	+	2	265	-	+	undefined	-	-
6	33/F	1 m	-	1:64	+	3	164	-	+	undefined	-	-
7	66/F	7 m	-	1:64	+	5	452	-	-	undefined	-	-
8	74/M	3 m	-	1:128	+	10	636	-	+	undefined	+	-
9	27/F	1 m	-	1:16	+	18	248	-	+	Early stage	+	-
10	55/M	6 m	-	1:128	+	14	2472	1:16	+	Late stage	+	-
11	57/M	1 m	-	1:64	+	14	410	-	+	Late stage	+	-
12	66/M	20 d	-	1:32	+	9	617	-	+	undefined	+	-
13	30/F	10 d	-	1:128	+	78	344	1:4	+	Early stage	+	Widespread dark red macules
14	63/F	1 m	-	1:128	+	189	1079	1:4	+	Undefined	+	-
15	43/F	4 m	-	1:64	+	3	348	-	+	Undefined	-	-
16	57/M	2 y	-	1:32	+	33	793	1:32	+	Late stage	+	-
17	45/F	6 y	-	1:4	+	1	411	-	-	Late stage	-	-

### Treatment and outcome

Fourteen patients were treated with benzylpenicillin sodium and three with ceftriaxone sodium after admission. At discharge, 12 patients perceived improvement of visual acuity, and visual acuity did not improve in five patients (Nos. 1, 10, 11, 16, and 17). Twelve patients returned to the ophthalmology department within 1 month or 1 year after discharge, with seven patients exhibiting significant improvement (Nos. 2, 3, 5, 8, 9, 13, and 14) and five patients exhibiting no significant improvement (Nos. 1, 7, 10, 11, and 15) (Table 1). In ten patients, serum and cerebrospinal exmination were improved during follow-up 6–12 months after treatment. Blood TRUST titer decreased by 4-fold in six cases, CSF-TRUST titer decreased 4-fold in three cases, and white blood cell count or trace protein decreased in six patients (Table 3).

**Table 3:** Comparison of laboratory examinations before and after treatment.

**case**	**pre-treatment**						**Post-treatment**					
	**Serum**		**Cerebrospinal fluid**				**Serum**		**Cerebrospinal fluid**			
	**TRUST**	**TPPA**	**Cells**	**Albumin**	**TRUST**	**TPPA**	**TRUST**	**TPPA**	**Cells**	**albumin**	**TRUST**	**TPPA**
			**(10^6^/L)**	**(mg/L)**					**(10^6^/L)**	**(mg/L)**		
1	1:8	+	8	620	-	+	1:8	+	1	355	-	+
3	1:256	+	15	296	-	+	1:1	+	-	-	-	-
6	1:64	+	3	164	-	+	1:4	+	-	-	-	-
8	1:128	+	10	636	-	+	1:4	+	3	440	-	+
9	1:16	+	18	248	-	+	1:2	+	5	406	-	+
10	1:128	+	14	2472	1:16	+	1:64	+	3	1062	1:1	+
13	1:128	+	78	344	1:4	+	1:64	+	4	356	1:1	+
14	1:128	+	189	1079	1:4	+	1:2	+	-	-	-	-
16	1:32	+	33	793	1:32	+	1:8	+	10	469	1:1	+
17	1:4	+	1	411	-	-	1:8	+	-	-	-	-

## Discussion

Ocular syphilis is easily misdiagnosed due to the lack of specific characteristics. Seven patients in the present study had previously been misdiagnosed. Misdiagnoses with other causes of uveitis, uveitis-induced retinitis, endogenous endophthalmitis, and diabetic oculomotor nerve palsy have also been reported (10, 11). Li et al. (12) analyzed potential reasons for misdiagnosis in 23 patients with ocular syphilis, concluding that misdiagnoses occur due to insufficient understanding of the clinical manifestations of eye disease, insufficient clinician recognition of ocular syphilis, and use of symptomatic treatments for non-specific ocular inflammatory lesions in the absence of laboratory screening for this etiology. In the present study, 16 of 17 cases (22 of 23 eyes) were identified as having visual acuity loss. This finding is comparable to other published studies. Jiang et al. (13) examined 41 eyes of 28 HIV-negative patients with ocular syphilis, in which the main complaints were blurry vision in 28 patients (100%), floaters in six patients (21.4%), visual field defect in five patients (17.9%), and ocular pain in five patients (17.9%). Moradi et al. (14) also demonstrated that >66.67% of the HIV-negative eyes and half of the HIV-positive eyes presented to their providers with visual impairment or blindness. For patients with decreased visual acuity and ocular neuropathy symptoms, clinicians should consider routine screening for syphilis to avoid misdiagnosis and delayed treatment.

Ocular syphilis can occur during any stage of syphilis infection. Tsuboi et al. (15) identified that 87.5% of patients with ocular syphilis developed disease within 2 years after syphilis infection, and that the median duration between syphilis infection and development of ocular symptoms was 11 months. They also proposed that if early syphilis patients have ocular symptoms, screening for ocular syphilis is necessary.

Any structure of the eye can be affected by syphilis, and Tsan et al. (16) updated various ocular diseases caused by *T. pallidum* infection. The most common manifestation of ocular syphilis is syphilitic uveitis (17), which accounts for <1–2% of total uveitis cases (16, 18). The proportion of uveitis caused by syphilis infection found in São Paulo hospitals increased from 1.88% in 1980 to 6.08% in 2014 (19). The proportion of syphilitic uveitis in France increased from 0.22% of all uveitis cases in 2012 to 2.5% in 2015 (20). Thus, the relative proportion of syphilitic uveitis is gradually increasing. Uveitis is classified as anterior uveitis, middle uveitis, posterior uveitis, and panuveitis. Anterior uveitis can present as superficial scleritis, scleritis, scleral keratitis, and iricliclitis, and posterior uveitis can present as chorioretinitis (focal or multifocal) and squamous chorioretinitis (16). The ocular manifestations of syphilis are generally non-specific, with exception to acute syphilitic posterior placoidchorioretinitis (ASPPC), a characteristic manifestation of ocular syphilis. ASPPC is characterized by the presence of one or more yellow-gray or yellow-white squamous lesions on the retina, usually located on or near the macula. This lesion is thought to be the result of active inflammation of the choroidal capillary-retinal pigment epithelial-retinal photoreceptor complex (21). Among the 17 cases in the present study, the most common manifestation was chorioretinitis, followed by optic neuropathy, and oculomotor nerve palsy in one case, the oculomotor nerve is the third cranial nerve, which innervates eye movement and levator muscle. When the oculomotor nerve is palsy, upper eyelid ptosis may occur, and the movement of the eye in, up and down is limited, resulting in extropia and diplopia. In this study, the ptosis of the right eye in case 17 was considered to be caused by *T. pallidum*. Similar reports have been made by several authors. The clinical manifestations of ocular syphilis are diverse and often lack specificity. Therefore, the diagnosis should be made by ophthalmic examination combined with serological testing for syphilis. Aspiration of ocular vitreous contents and testing aqueous humor with *T. pallidum*-specific antibody assays or by PCR detection can also be used (22, 23). In the present study, the detection of specific antibodies to *T. pallidum* in the aqueous humor of case No. 13 and the calculation of Goldman-Whitmer coefficient (GWC) also provided a basis for the diagnosis of ocular syphilis. Aqueous humor GWC is also a diagnostic criteria for ocular syphilis (5). GWC ≥4 indicates that the amount of *Treponema*-specific antibodies in the aqueous humor significantly exceeds circulating *Treponema-*specific antibodies, which in consideration of the blood-eye barrier suggests the antibody is produced *in situ* by ocular tissues (24, 25). Ocular syphilis is in some cases associated with neurosyphilis, both of which can develop at any stage of syphilis infection. Some investigators have proposed that specific *T. pallidum* strains are neuropathogenic, but it is not clear whether specific *T. pallidum* strains cause ocular infection. Blandine Gutierrez et al. (26) propose that the retina and optic nerve are extensions of the central nervous system, so optic nerve retinitis is considered to be an extension or warning sign of neurosyphilis. Sixty per cent of ocular syphilis patients have abnormal CSF, which is more likely in HIV-positive patients with ocular syphilis (27). Thus, the CDC recommends that all patients with ocular syphilis be tested for CSF abnormalities and HIV (7). A study of 326 syphilis patients revealed that patients with serum titers ≥1:32 were more likely to develop neurosyphilis (28). Jing et al. (29) also proposed that CSF examination should be performed to screen for neurosyphilis if the disease course was unknown and serum titers were high ≥1:32. Ting et al. (30) found that CSF abnormalities were more likely in ocular syphilis patients with TRUST titer ≥1:32. All cases in the present study underwent lumbar puncture for CSF examination and serum HIV antibody testing. Among the 11 cases that met the diagnostic criteria for neurosyphilis, nine cases had titers ≥1:32, which was consistent with previous reports. These findings underscore the need for CSF testing in patients with high titers. The first choice of treatment for ocular syphilis or neurosyphilis is (8) intravenous penicillin (18–24 million units) daily for 10–14 days, followed by three weekly intramuscular benzathine penicillin injections (24 million units). Patients allergic to penicillin can be treated with daily intravenous ceftriaxone (2 g) for 10–14 days. After completion of treatment, patients with abnormal CSF should be followed up every 6 months until CSF returns to normal. Glucocorticoids prevent the Jarisch-Herxheimer reaction and alleviate ocular or neurological manifestations. Topical corticosteroid eye drops can effectively treat interstitial keratitis and anterior uveitis. Oral and intravenous corticosteroids can be used to treat posterior uveitis, scleritis, and optic neuritis. Importantly, only early, full-course, and systematic administration of antibiotics can cure the primary disease of syphilis. Neurotrophic drugs can enhance the uptake and utilization of oxygen and glucose in cells, which is beneficial to the recovery of neurological function (31). NaXin et al. (32) used mecobalamin as adjuvant therapy in 98 patients with ischemic optic neuropathy, and the results showed that the therapeutic effect was better than that of patients with single drug. Dongping Zheng et al. (33) used mecobalamin as adjuvant therapy in the study of central retinal artery occlusion, and the results also showed that the therapeutic effect was better than that of single drug patients. In the present study, visual acuity improved in 12 patients and was unchanged at discharge in five patients. After discharge, 12 patients followed up with the ophthalmology clinic for ophthalmic examinations, of which 7 patients had significantly improved visual acuity, the course of these 7 patients was 10 days to 3 months, the other 5 patients did not improve visual acuity, the course of these 5 patients was 1 month to 1 year, we consider the ocular symptoms in patients with the shorter the time, their visual acuity improvement could be better. This also suggests that early diagnosis and treatment of ocular syphilis may be helpful to the recovery of vision.

Ocular syphilis can easily be misdiagnosed as other ocular diseases in clinical practice, delaying treatment and eventually causing irreversible ocular damage. Therefore, both ophthalmologists?dermatologists and general practitioners must be aware of the eye diseases caused by *Treponema pallidum* infection. In the present study, almost all cases had clinical manifestations of visual acuity loss, suggesting visual acuity loss is an early warning indicator for ocular syphilis screening. In these cases, CSF testing should be performed to screen for neurosyphilis as soon as possible to avoid future development of neurological or psychiatric symptoms. The present study has shortcomings that should be considered in its interpretation, including the limited number of cases, incomplete examination data, and insufficient follow-up period. To further support these findings, larger, prospective studies are needed.

## Data availability statement

The original contributions presented in the study are included in the article/supplementary material, further inquiries can be directed to the corresponding authors.

## Ethics statement

The studies involving human participants were reviewed and approved by the Ethics Committee of The Fifth People's Hospital of Suzhou. The patients/participants provided their written informed consent to participate in this study.

## Author contributions

All authors listed have made a substantial, direct, and intellectual contribution to the work and approved it for publication.

## Funding

This work was supported by Suzhou Clinical Medical Expert Team Introduction Project (SZYJTD201811), Science and Technology Project of Suzhou Health and Family Planning Commission (LCZX201818), Suzhou Science and Education Revitalizing Health Youth Science and Technology Project (KJXW2021052), and Suzhou Science and Technology Plan Project (SKJY2021137).

## Conflict of interest

The authors declare that the research was conducted in the absence of any commercial or financial relationships that could be construed as a potential conflict of interest.

## Publisher's note

All claims expressed in this article are solely those of the authors and do not necessarily represent those of their affiliated organizations, or those of the publisher, the editors and the reviewers. Any product that may be evaluated in this article, or claim that may be made by its manufacturer, is not guaranteed or endorsed by the publisher.
